# Environmental-demographic determinants associated with tuberculosis prevalence in seven African countries: an aggregated dataset analysis

**DOI:** 10.1016/j.eclinm.2026.103773

**Published:** 2026-01-28

**Authors:** Tessa M.I. Haverkate, Daniella Brals, Egbal A.B. Abukaraig, Nathan Kapata, Pascalina Kapata-Chanda, Bruce Kirenga, Eveline Klinkenberg, Irwin Law, Llang B. Maama-Maime, Sizulu Moyo, Joshua Obasanya, Elizeus Rutebemberwa, Logan Stuck, Edine Tiemersma, Frank Cobelens

**Affiliations:** a Amsterdam UMC, Location University of Amsterdam, Department of Global Health, Amsterdam Institute for Global Health and Development, Amsterdam, the Netherlands; b Department of Public Health, Erasmus MC, University Medical Center Rotterdam, Rotterdam, the Netherlands; c Research and Development Centre, Alfjr College for Science and Technology, Khartoum, Sudan; d Ministry of Health, Lusaka, Zambia; e Makerere University Lung Institute & Division of Pulmonary Medicine, Department of Medicine, Makerere University College of Health Sciences, Kampala, Uganda; f Connect TB, The Hague, the Netherlands; g Global Programme on Tuberculosis and Lung Health, World Health Organization, Geneva, Switzerland; h Ministry of Health National TB and Leprosy Programme, Maseru, Lesotho; i Human Sciences Research Council, Cape Town, South Africa; j Nigeria Centre for Disease Control, Abuja, Nigeria; k Department of Health Policy, Planning and Management, School of Public Health, Makerere University, Kampala, Uganda; l Wageningen University & Research, Wageningen, Netherlands; m KNCV Tuberculosis Foundation, The Hague, the Netherlands

**Keywords:** Tuberculosis, Ecological, Environmental, Sociodemographic, Planetary health

## Abstract

**Background:**

Knowledge about environmental and demographic determinants of tuberculosis is largely limited to studies with ecological designs. We explored the association between these determinants and tuberculosis prevalence in an individual participant dataset aggregated across seven African countries.

**Methods:**

Data of nationally representative tuberculosis prevalence surveys (2012–2019) from highly endemic countries were supplemented with publicly accessible data at district level. Associations between individual-level diagnosis of bacteriologically confirmed tuberculosis and district-level environmental-demographic variables were investigated in generalised linear mixed-effects models accounting for the multi-level structure of the data.

**Findings:**

Of 322,615 participants aged ≥15 years across 400 districts, 976 were newly diagnosed with tuberculosis (prevalence 183–638/100,000 across the countries). Living at latitude 7.6–14.6° (adjusted odds ratio, aOR 2.07, 95% confidence interval, 95% CI 1.48–2.90) or in higher population density (aOR 1.07 per percent increase in mean population density, 1.01–1.13), or urban districts (aOR 1.31, 1.11–1.54) were independently associated with higher prevalence. Living in distsricts above 900 m altitude (aOR 0.52, 0.32–0.84), with 50–100 mm precipitation (aOR 0.62, 0.46–0.84), or at higher temperature (aOR 0.93 per degree Celsius, 0.88–0.98) was independently associated with lower tuberculosis prevalence. No significant associations were observed with fine particulate matter (aOR 1.04, 0.70–1.54 for 20–40 μg/m^3^, 0.82, 0.44–1.53 for >40 μg/m^3^), solar radiation (aOR 1.04, 0.93–1.15) or International Wealth Index (aOR 1.01 (1.00–1.02).

**Interpretation:**

Our results suggest that in high-burden African countries, some of the variation in tuberculosis prevalence can be explained by environmental and demographic factors that merit further investigation.

**Funding:**

Mr Willem Bakhuys Roozeboom Foundation.

**Research in context**

**Evidence before this study**

The potential impact of environmental and demographic variables on tuberculosis (TB) prevalence and outcomes has gained attention in recent years, reflecting a growing recognition of the complex interplay between such factors and health outcomes. We searched PubMed with the search string “(“tuberculosis” OR “TB”) AND (“ecological” OR “environmental” OR “outdoor air pollution”)” for publications from January 1, 1990, to August 20, 2025. Additionally, each ecological determinant (altitude, latitude, particulate matter with aerodynamic diameter ≤2.5 μm (PM_2.5_), population density, precipitation, solar radiation, temperature, and urbanisation) was searched separately in combination with tuberculosis/TB in the title (e.g., (tuberculosis [Title]) AND (altitude [Title])). Our search identified a limited number of studies related to tuberculosis and ecological variables; the majority focussed on the seasonality or geospatial distribution of tuberculosis. Significant associations were found between tuberculosis and various ecological factors, including altitude, latitude, concentration of PM_2.5_, population density, precipitation, sunshine duration, and temperature. However, the data disproportionately focused on Asia, particularly China; few studies were conducted in Africa. Moreover, existing studies generally relied on non-representative samples of clinic attendees in limited geographic areas. Where representative surveys were used, these were typically single-country studies with relatively few TB cases. Studies that combined data from multiple countries generally did so using aggregate prevalence estimates rather than individual-level data, risking ecological fallacy. A recent study by Liyew et al. (2025) attempted to address these questions across African countries, but was based on aggregated prevalence estimates rather than individual participant data, and therefore faced similar limitations.

**Added value of this study**

We comprehensively examined the environmental-demographic determinants of tuberculosis prevalence across seven highly endemic countries in Africa, incorporating substantial geographic heterogeneity. The selection of ecological variables in our study was guided both by prior literature suggesting plausible associations with tuberculosis, and by the availability of structured datasets that allow consistent linkage across multiple national prevalence surveys. By using data from 322,615 adults across seven nationally representative surveys with standardised protocols, our study benefits from a large and representative sample. The supplementation with a variety of structured environmental-demographic data obtained from publicly accessible datasets further enriched the data allowing for more precise estimates of the associations between environmental-demographic determinants and tuberculosis prevalence. Our study reveals that in high-burden African regions, TB prevalence is closely linked to geographic determinants, in particular altitude, latitude, precipitation and temperature, and sociodemographic determinants, in particular density and urbanisation.

**Implications of all the available evidence**

Our study reveals significant environmental-demographic determinants of tuberculosis prevalence in African settings, adding new evidence to previous findings predominantly from Asia. By aggregating data from seven geographically heterogeneous countries, we provide more robust estimates. The integration of these determinants into public health strategies could allow for more precise targeting of high-risk districts, potentially improving resource allocation and intervention timing. Additionally, the variability observed across regions suggests that longitudinal monitoring of ecological shifts—such as seasonal changes in precipitation and cumulative exposure to air pollutants—may be crucial to understanding tuberculosis trends. Further studies with more granular and temporally specific data are needed to better understand the full scope of the ecological impact on tuberculosis transmission and control.

## Introduction

With an estimated 10.8 million people falling ill and 1.25 million dying each year, tuberculosis (TB) remains a major global health problem; Africa is among the global regions with the highest burden.^1^ The etiologic agent *Mycobacterium tuberculosis* (*Mtb*) has infected an estimated quarter of the global population.^2^ Only a minority of those infected progress to disease. Several individual-level risk factors for TB disease have been documented, including HIV coinfection, malnutrition, diabetes, excessive alcohol use and tobacco smoking.^3^, ^4^, ^5^ In addition, there are potential environmental and demographic determinants of TB disease that operate at population level such as altitude, latitude, air quality, climate and urbanicity, and can vary widely across regions and populations.^6^, ^7^, ^8^, ^9^

These determinants could affect TB incidence through biological pathways, as well as through social mechanisms. For example, geographic and weather-related factors, such as altitude, latitude, and seasonal variations, may affect TB transmission dynamics by influencing oxygen tension, seasonal changes in immune function, and time spent indoors.^10^ Moreover, ecological determinants may contribute to social stratification and inequalities in access to resources.^8^^,^^11^ Research into these determinants has however been limited to local studies, while those that were done across settings have been hampered by being based on notified cases (i.e. not accounting for access to care),^12^, ^13^, ^14^, ^15^, ^16^ lack of population representativeness,^7^^,^^10^^,^^17^ between-study differences in case detection methods and TB case definitions,^7^^,^^10^^,^^17^^,^^18^ or only using aggregated data with substantial risk of ecological fallacy.^7^^,^^12^^,^^13^^,^^17^^,^^19^ Moreover, most studies were done in Asia,^12^^,^^14^, ^15^, ^16^^,^^20^, ^21^, ^22^, ^23^ whereas few focussed on the African continent^24^^,^^25^ that bears one of the largest TB burdens.^1^

Aggregating data from nationally representative TB prevalence surveys (TBPS) has the potential to overcome these shortcomings. Since 2000, the World Health Organization (WHO) has supported national TBPS in high-burden countries to reliably estimate incidence and mortality.^26^ Between 2012 and 2019, thirty surveys have been conducted globally, of which 17 in Africa, that deployed similar designs, case detection methods and TB case definitions, offering the opportunity for valid comparison of environmental and demographic determinants of TB disease across countries and settings.^26^

We analysed individual participant data (IPD) in an aggregated dataset of TBPSs from highly endemic African countries and supplemented those data with district-level information to investigate the association between environmental and demographic determinants and TB prevalence while accounting for individual-level variation in TB risk.

## Methods

### Datasets

We used IPD of seven nationally representative TBPS conducted between 2012 and 2019 in Ghana, Lesotho, Nigeria, South Africa, Sudan, Uganda, and Zambia. These countries had been included in a systematic review and IPD meta-analysis (IPDMA) to quantify the contribution to TB prevalence of subclinical TB following different case definitions.^27^ This systematic review had sought IPD from all 17 African TBPS conducted between 2012 and 2019, but failed obtain them for nine surveys due to responsible staff being no longer in their position or lack of approval for data sharing. Of the remaining eight datasets, one was excluded due to the absence of administrative levels (The Gambia).

All included surveys had been done under the auspices of the WHO following a standard protocol, and applied multistage cluster sampling, standardized case detection methods and similar bacteriological outcome definitions (Supplementary Table S1). Within districts, villages or communes were selected using probability proportional to size sampling. Households within the selected clusters were visited, and individuals aged 15 and older who were present were invited to a central survey location. Screening for presumptive TB was conducted using symptom assessment and chest X-rays (CXR). Generally, participants presenting symptoms such as persistent cough lasting a minimum of two weeks and/or showing abnormalities on CXR were classified as screen positive and eligible for further sputum examination. As surveys used different bacteriological confirmation methods, we applied a standardized definition for prevalent pulmonary TB across all datasets, which was as culture- or GeneXpert MTB/Rif (Xpert)-positive sputum (exact algorithm reflected in Supplementary Table S1) in individual participants who had not received TB treatment prior to the TBPS for 30 days or more.

### Environmental-demographic determinants

A range of environmental-demographic determinants of TB was considered in the current analysis, including altitude, fine particulate matter (particulate matter with aerodynamic diameter ≤2.5 μm, PM_2.5_), latitude, population density, precipitation, solar radiation, temperature, and urbanisation. Thereto, survey data were supplemented using Linked Open Data; structured data released under an open licence (Table 1). The Database of Global Administrative Areas (GADM) and Humanitarian Data Exchange (HDX) were sourced to link administrative levels available from the IPD to spatial boundaries.^28^, ^29^, ^30^, ^31^ The IPD included the administrative unit name (level 1 and 2) from which the participant was sampled. Data linkage was performed whereby each individual was assigned the spatial attributes corresponding at administrative level 2 where available, or otherwise at the highest available level (level 1 in Sudan). This level is hereafter referred to as “district”. Where necessary, administrative names were harmonised between datasets to accounts for differences in spelling, diacritics or formatting. NASA Socioeconomic Data and Applications Centre (SEDAC) combines Aerosol Optical Depth retrievals from multiple satellite algorithms and was referred to for the mean concentration of PM_2.5_.^32^^,^^33^ WorldClim version 2.1 was used to extract data on monthly meteorological variables.^34^ For data obtained from SEDAC and WorldClim raster values within each district polygon, as defined by the GADM/HDX boundaries, were extracted and averaged to produce a single mean value per district. WorldPop data, which utilises official United Nations population estimation censuses and a geospatial data set to disaggregate them to counts for each square kilometre classified as settled by humans, were used to determine population density.^33^ A deterministic linkage process was applied based on administrative level unit names. Finally, to compare socioeconomic status across countries the International Wealth Index (IWI) was extracted from Global Data Lab, for which deterministic data linkage was done based on administrative level 1 name in IPD.^35^^,^^36^

**Table 1:** Overview of determinants averaged over district-level included in the analytical dataset, their data source and definition.

Determinant	Data source	Extracted data
Altitude	GADM/HDX^28^, ^29^, ^30^, ^31^	District's altitude in m above sea level averaged over district-level.
Latitude	GADM/HDX^28^, ^29^, ^30^, ^31^	Absolute latitude angle (ϕ) in degrees north or south of the equator averaged over district-level.
Precipitation	WorldClim version 2.1^34^	Annual average precipitation in mm between 1970 and 2000 averaged over district-level.
PM_2.5_	SEDAC^32^	Annual average concentrations in μg/m^3^ of all composition ground-level PM_2.5_ in the year of the survey averaged over district-level.
Population density	WorldPop^33^	Number of people per km^2^ in the year of the survey averaged over district-level.
Solar radiation	WorldClim version 2.1^34^	Annual average solar radiation in Kilojoule/m^2^/day between 1970 and 2000 averaged over district-level.
Temperature	WorldClim version 2.1^34^	Annual average temperature in degrees Celsius between 1970 and 2000 averaged over district-level.
Urbanisation	IPD^27^	Urbanisation defined based on administrative classification provided in the IPD. Districts classified as “Peri-Urban”, “Semi-Urban”, or “Urban” were grouped as urban, while those classified as “Rural” were grouped as rural.
International Wealth Index	Area Database of the Global Data Lab^35^^,^^36^	Asset based wealth index in year of the survey averaged over sub-regional level (GADM level 1).

Abbreviations used: GADM = Database of Global Administrative Areas, HDX = Humanitarian Data Exchange m = metres, mm = millimetres, PM_2.5_ = particulate matter with aerodynamic diameter ≤2.5 μm, (k)m^2^ = square (kilo)metre, μg/m^3^ = micrograms per cubic metre air, SEDAC = NASA Socioeconomic Data and Applications Centre, IPD = Individual Participant Data.

### Statistical analysis

The IPD of the 7 studies were assembled into a single dataset, recoding variables as needed. Included in the analyses were all persons participating in the surveys. Excluded were all whose district of residence could not be matched to the GADM/HDX database (Supplementary Table S2). The unit of analysis was the individual participant. The outcome measure was the individual's diagnosis of TB.

Descriptive statistics were generated to assess the distribution of TB prevalence and the independent variables across all districts. To ensure variable linearity, residual plots were examined, and log transformation or categorisation was applied to non-linear variables. Univariate and multivariate relationships between TB prevalence and each independent variable were examined using generalised linear mixed-effects models. A multilevel logistic regression model (generalized linear mixed model, GLMM) with a binomial distribution and logit link function was fitted to assess the association between the individual-level diagnosis of TB (binary outcome) and environmental-demographic predictors (Supplementary Equation E1).

These models handle data at different levels by incorporating both fixed effects, which account for the overall impact of predictor variables, and a random intercept for country level was included to account for clustering at the country level and unobserved heterogeneity. Additionally, the multivariate model was adjusted for individual participant age and sex. The variance and standard deviation of the random intercept were estimated to quantify between-country variability. Variance Partition Coefficient (VPC) was calculated to express the proportion of total variance attributable to differences between countries, using the latent variable method for logistic models. Variance inflation factors (VIF) were calculated for all covariates. Variables with VIF >10 were considered highly collinear. Sensitivity analyses were performed by omitting each high-VIF variable in turn and re-estimating the model to assess robustness of effect estimates. Interactions between sex and each of altitude, latitude, precipitation, and temperature, between population density and urbanisation, and between latitude and temperature were assessed in secondary analyses. To explore whether the association between population density and TB prevalence differed by urbanisation level, stratified analyses were conducted separately for urban and rural areas. All statistical analyses were conducted using the ‘lme4’ package in R 4.2.2.^37^

### Ethics

All surveys from which the datasets were included in this analysis had received ethical approval from their respective national bodies. The present analysis was based entirely on existing anonymised datasets, therefore no separate ethical review was sought.

### Role of the funding source

The funder of the study had no role in study design, data collection, data analysis, data interpretation, or writing of the report.

## Results

**Fig. 1:**
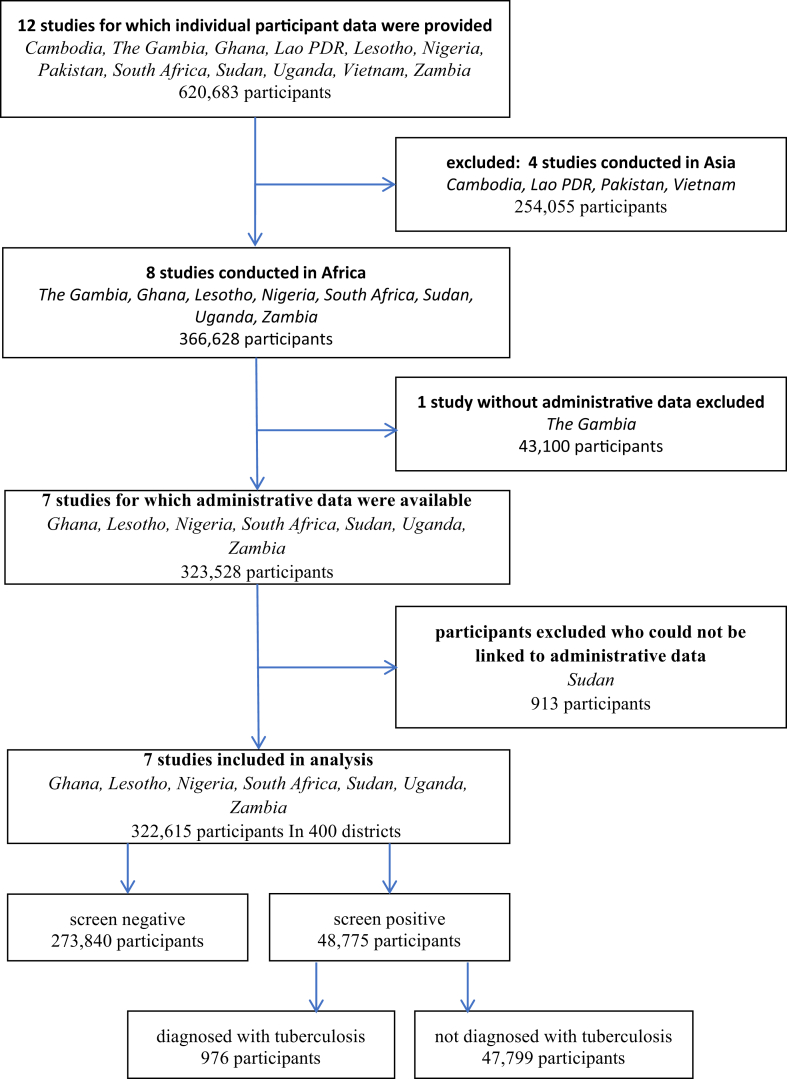
Study selection, including numbers of participants.

Data from 322,615 participants divided over 400 districts across seven countries were used in this study (Fig. 1, Table 2). Of those, 976 were newly diagnosed with TB (Supplementary Figure S1). Just over half (58.0%) were male, the mean age was 36.5 years (standard deviation 17.3). The average district altitudes ranged between 4 m and 2883 m. All districts but two (in South Africa) were exposed to annual average PM_2.5_ concentrations exceeding the WHO annual Air Quality Guidelines level of 5 μg/m^3^.^38^ Most districts, except those in South Africa, were located between the Tropic of Cancer and the Tropic of Capricorn. The average temperature across the districts ranged from 7.8 to 30.5 °C. Population density varied between 2 and 69,832 people per km^2^. High correlation was found between temperature and altitude, and precipitation and solar radiation (Supplementary Figure S2). These correlations were not adjusted for in the main analysis. No strong correlations were observed between the other variables.

**Table 2:** Sample description, stratified by tuberculosis diagnosis.

Characteristics	Screen Positive (n = 48,775)		Screen negative (n = 273,840)	Total (n = 322,615)
	Diagnosed with tuberculosis (n = 976)	Not diagnosed with tuberculosis (n = 47,799)		
**Individual determinants**				
Sex, n (%)				
Male	566 (58.0)	19,278 (40.3)	112,980 (41.3)	132,824 (41.2)
Age group (years), n (%)				
15–24	119 (12.2)	7866 (16.5)	91,311 (33.3)	99,296 (30.8)
25–34	216 (22.1)	8705 (18.2)	63,646 (23.2)	72,567 (22.5)
35–44	193 (19.8)	7739 (16.2)	46,939 (17.1)	54,871 (17.0)
45–54	146 (15.0)	7033 (14.7)	32,698 (11.9)	39,877 (12.4)
55–64	121 (12.4)	6487 (13.6)	20,388 (7.4)	26,996 (8.4)
65–74	85 (8.7)	4529 (9.5)	9274 (3.4)	13,888 (4.3)
≥75	74 (7.6)	4199 (8.8)	6334 (2.3)	10,607 (3.3)
NA	22 (2.25)	1241 (2.6)	3250 (1.2)	4513 (1.4)
**Environmental-demographic determinants**				
Altitude (m), n (%)				
≤450	316 (32.4)	16,193 (33.9)	114,642 (41.9)	131,151 (40.7)
>450, ≤900	142 (14.5)	12,280 (25.7)	54,175 (19.8)	66,597 (20.6)
>900	518 (53.1)	19,326 (40.4)	105,023 (38.4)	124,867 (38.7)
Latitude (absolute degrees), n (%)				
≤7.6°	301 (30.8)	11,511 (24.1)	95,839 (35.0)	107,651 (33.4)
>7.6, ≤14.6°	346 (35.5)	21,779 (45.6)	124,912 (45.6)	147,037 (45.6)
>14.6°	329 (33.7)	14,509 (30.4)	53,089 (19.4)	67,927 (21.1)
Precipitation (mm), n (%)				
0	177 (18.1)	11,536 (24.1)	76,756 (28.0)	88,469 (27.4)
>0, ≤50	501 (51.3)	23,736 (49.7)	126,125 (46.1)	150,362 (46.6)
>50	291 (29.8)	12,237 (25.6)	68,970 (25.2)	81,498 (25.3)
NA	7 (0.7)	290 (0.6)	1989 (0.7)	2286 (0.7)
PM_2.5_ (μg/m^3^), n (%)				
≤20	513 (52.6)	19,607 (29.4)	91,788 (33.5)	111,908 (34.7)
>20, ≤40	156 (16.0)	14,046 (29.4)	76,169 (27.8)	90,371 (28.0)
>40	304 (31.1)	13,699 (28.7)	104,167 (38.0)	118,170 (36.6)
NA	3 (0.3)	447 (0.9)	1716 (0.6)	2166 (0.7)
Population density, mean number of people per km^2^ (sd)	1584 (4872)	1230 (4379)	1645 (5429)	1584 (5287)
Solar radiation (MJ/m^2^/day), mean (sd)	18.0 (3.3)	19.0 (3.3)	18.7 (2.6)	18.7 (2.8)
Temperature (degrees Celsius), mean (sd)	20.8 (5.8)	21.6 (5.5)	22.5 (5.0)	23.0 (5.1)
Urbanisation, n (%)				
Urban	518 (53.1)	19,459 (40.7)	126,054 (46.0)	146,031 (45.3)
International Wealth Index, mean (sd)	46.6 (17.6)	43.4 (17.5)	41.7 (16.5)	42.0 (16.7)

Abbreviations used: NA = not available, m = metres, mm = millimetres, PM_2.5_ = particulate matter with aerodynamic diameter ≤2.5 μm, μg/m^3^ = micrograms per cubic metre air, km^2^ = square kilometre, sd = standard deviation, MJ/m^2^/day = Megajoule per square metre per day.

Distribution of environmental and sociodemographic characteristics among all participants diagnosed with tuberculosis (n = 976), culture negative participants (n = 47,799), and the total sample (n = 322,615). Values listed as counts unless otherwise indicated. Values in parentheses represent percentages of participants, calculated relative to the column total indicated at the top of each column (e.g., percentage of the Total (n = 322,615)), unless otherwise specified (e.g., mean (sd)).

In the generalised linear mixed-effects model, latitude, population density, and urbanisation were positively associated with TB prevalence at individual level. Those living in districts with absolute latitude between 7.6 and 14.6° had 2.07 times higher odds of TB than those in districts with lower latitude (95% confidence interval, 95% CI 1.48–2.90). An increase of one percent in mean population density equalled an increase of 1.07 in TB prevalence (95% CI 1.01–1.13). Those living in an urban district had 1.31 times greater odds of TB than those living in rural districts (95% CI 1.11–1.54). In addition, males and those aged 25–34 and 35–44 years had independently increased odds of TB compared to females and those aged 15–24 years respectively. Altitude, precipitation, and temperature showed negative association with TB prevalence (Table 3). The odds of TB for those living at altitude above 900 m was 0.52 (95% CI 0.32–0.84) times the odds of those at altitude <450 m. The odds of TB for those living at in a district with annual precipitation between 50 and 100 mm was 0.62 times the odds (95% CI 0.46–0.84) those in a district with <50 mm. The odds of TB was 0.93 times smaller for each degree Celsius increase in mean temperature (95% CI 0.88–0.98). The variance of the random intercept for the country-level clustering was estimated at 0.63 (sd = 0.80), indicating substantial heterogeneity between countries. The VPC was 0.16, suggesting that approximately 16% of the total variance in TB prevalence was attributable to differences between countries.

**Table 3:** Odds ratio and 95% confidence interval for tuberculosis prevalence in 322,615 individuals in Ghana, Lesotho, Nigeria, Sudan, South-Africa, Uganda, and Zambia.

	Univariate analysis		Multivariate analysis	
	Odds ratio (95% CI)	P-value	Odds ratio (95% CI)	P-value
**Environmental-demographic variables**				
Altitude (m)				
≤450 m	ref			
>450, ≤900 m	0.67 (0.48–0.91)	0.01	0.79 (0.56–1.14)	0.21
>900 m	0.55 (0.37–0.83)	0.004	0.52 (0.32–0.84)	0.01
Latitude (absolute degrees)				
≤7.6°	ref			
>7.6, ≤14.6°	1.34 (1.05–1.71)	0.01	2.07 (1.48–2.90)	<0.01
>14.6°	1.13 (0.77–1.65)	0.52	1.48 (0.90–2.44)	0.13
Precipitation (mm)				
>0, ≤50	ref			
>50, ≤100	0.82 (0.67–1.01)	0.06	0.62 (0.46–0.84)	0.002
>100	0.82 (0.57–1.18)	0.29	0.95 (0.65–1.38)	0.78
PM_2.5_ (μg/m^3^)				
≤20	ref			
>20, ≤40	1.17 (0.83–1.66)	<0.01	1.04 (0.70–1.54)	0.85
>40	0.96 (0.48–1.84)	0.19	0.82 (0.44–1.53)	0.52
Population density, logarithm of mean people per km^2^	1.09 (1.05–1.13)	<0.01	1.07 (1.01–1.13)	0.02
Solar radiation (MJ/m^2^/day)	0.94 (0.88–1.01)	0.07	1.04 (0.93–1.15)	0.50
Temperature (degrees Celsius)	0.96 (0.93–1.00)	0.03	0.93 (0.88–0.98)	0.01
Urbanisation				
Rural	ref			
Urban	1.59 (1.39–1.81)	<0.01	1.31 (1.11–1.54)	0.001
International Wealth Index	1.02 (1.01–1.03)	<0.01	1.01 (1.00–1.02)	0.10
**Individual variables**				
Sex				
Female	ref			
Male	1.89 (1.66–2.15)	<0.01	1.82 (1.60–2.08)	<0.01
Age group (years)				
15–24	ref			
25–34	1.59 (1.27–2.00)	<0.01	1.58 (1.26–1.99)	<0.01
35–44	1.53 (1.22–1.93)	<0.01	1.48 (1.17–1.87)	0.001
45–54	1.21 (0.95–1.55)	0.13	1.19 (0.93–1.52)	0.17
55–64	1.07 (0.83–1.39)	0.59	1.08 (0.83–1.39)	0.58
65–74	1.02 (0.77–1.35)	0.90	1.01 (0.76–1.35)	0.92
≥75	0.99 (0.73–1.32)	0.92	1.05 (0.78–1.42)	0.73

Abbreviations used: CI 95% = 95% confidence interval, m = metres, ref = reference category, mm = millimetres, PM_2.5_ = particulate matter with aerodynamic diameter ≤2.5 μm, μg/m^3^ = micrograms per cubic metre air, km^2^ = square kilometre, MJ/m^2^/day = Megajoule per square metre per day, sd = standard deviation.

Association between newly diagnosed with tuberculosis with environmental-demographic determinants in individuals from the total sample from Ghana, Lesotho, Nigeria, South Africa, Sudan, Uganda, and Zambia (n = 322,615). The variance of the random intercept for the country-level clustering = 0.63 (sd = 0.80). Variance Partition Coefficient = 0.16.

Sensitivity analyses and secondary models did not alter the direction or magnitude of the main associations, supporting the robustness of the primary findings. Collinearity assessment revealed high VIFs for latitude (11.84) and PM_2.5_ (10.33), indicating strong correlation between these variables. Removal of PM_2.5_ did not materially change the results, and latitude remained significant (Supplementary Table S3). Removal of latitude had little effect on most coefficients, but population density lost statistical significance, suggesting some dependency between latitude and population density. Significant interaction was found between both categories of latitude and sex (P < 0.01, P = 0.01). For higher absolute latitude, being a male compared to a female had less effect on TB prevalence. No significant interactions were found between sex and altitude, precipitation or temperature, between population density and urbanisation, and between latitude and temperature. Exploratory stratified analyses by urbanisation level indicated that the overall patterns and effect directions were consistent across strata, and there was no indication that urbanisation fully accounted for the effect of population density was no indication that urbanisation fully accounted for the effect of population density.

## Discussion

Our analysis of an aggregated dataset of 7 nationally representative surveys from Africa revealed the role of environmental-demographic factors in patterns of TB prevalence. Latitude, population density, and urbanisation emerged as significant positive correlates, pointing to the heightened TB prevalence in areas at 7.6 and 14.6° absolute latitude, densely populated areas, and urban centres. In contrast, altitude above 900 m, precipitation between 50 and 100 mm, and higher temperature were inversely associated with TB prevalence, suggesting potential direct or indirect protective effects.

Previous studies have suggested an inverse association between altitude and TB but have rarely been able to disentangle the effect of altitude itself from other characteristics such as population density.^7^^,^^10^^,^^17^^,^^39^ Given that in vitro growth of *Mtb* is enhanced by high oxygen tension, one hypothesis to explain our findings is that the decrease of barometric pressure as altitude increases leads to lower alveolar oxygen pressure, hindering the development of tuberculous lesions.^39^ Our analyses have also revealed significant negative TB prevalence associations for precipitation and temperature. Contrary to our findings, recent systematic review based studies found a positive correlation between precipitation and TB risk.^10^^,^^17^ Qin et al. (2022) suggest that heavy rainfall may increase time spent indoors, thereby elevating the risk of *Mtb* transmission due to crowding and poor ventilation.^10^ Additionally, disruptions to healthcare access during periods of heavy precipitation may affect TB outcomes. The discrepancy between their findings and ours may relate to the relatively narrow range of precipitation observed in our study region, which primarily experienced low levels of rainfall compared to other parts of the world.^40^ This limited range may have restricted our ability to accurately capture the full relationship between precipitation and TB. In addition, both systematic reviews may have suffered from bias as they included studies that were not population-representative without standardization of case finding approaches and TB disease definitions.^10^^,^^17^

Altitude, latitude, temperature, and precipitation can all affect exposure to ultraviolet B (UV-B). Increased exposure to UV-B, such as at higher altitudes or during low precipitation, increases the generation of vitamin D in the skin.^19^ By modulating the function of macrophages, 1.25-dihydroxyvitamin D, the active form of vitamin D, helps reduce the intracellular growth of *Mtb*.^19^ In addition to indirectly enhancing the innate immune response, UV-B may also suppress adaptive immunity while enhancing innate immunity.^41^ A role of vitamin D in the geographic variation in TB prevalence is also suggested by the independent associations we found with latitude. Evidence from Chile and India further supports the relationship between latitude and TB prevalence, showing a consistent global pattern.^12^^,^^13^ We observed a near–significant association between precipitation and solar radiation, likely driven by the reduced solar radiation with clouds covering the sun during continuous heavy rain. However, we did not observe correlations between altitude and solar radiation, nor between latitude and solar radiation. This may be due to the use of averaged values at district-level in our study, rather than finer geographical units or individual-level data. Averaging altitude, latitude, and temperature may have weakened potential correlations with solar radiation, while using annual average solar radiation likely fails to capture seasonal UV-B shifts.^19^ Moreover, mountain ranges, valleys, and other terrain features can create microclimates, leading to variations in solar exposure even within districts with similar altitudes and latitudes.

We found that TB prevalence was associated with urban residence, even after adjustment for population density, which is a known sociodemographic TB risk factor.^42^^,^^43^ This suggests that urban environment by itself increases TB incidence, its duration, or both. These urban-rural disparities are consistent with findings from other regions.^43^ Contributing factors could include higher HIV prevalence in urban areas, poor living conditions and access to diagnosis and treatment–particularly in settlements of rural-urban migrants that increasingly make up large parts of African urban areas.^44^, ^45^, ^46^

This study had limitations. Not all available IPD could be included.^27^ The study could not capture the full range of geographic and environmental heterogeneity present in the dataset, restricting the generalisability of the findings. We did not have details on HIV status, smoking or individual nutritional status, nor did we have data on household-level socio-economic position, are all established risk factors for TB incidence or prevalence. Our study may therefore have failed to identify additional social determinants, and our findings may suffer from residual confounding. Although several surveys included household asset scores, these are relative measures of wealth and cannot be used for cross-country comparisons.^47^ We therefore included the IWI that measures the economic situation of households using asset data from 165 surveys held over a period of 15 years in 97 countries across the world.^36^ While this index ensures comparability across countries, the geographic granularity of the IWI is less than individual household measurement, which could have resulted in residual confounding by socio-economic status. As access to and quality of healthcare are key determinants of TB diagnosis and treatment, the potential for confounding by district healthcare access and quality is a concern, possibly obscuring the true relationship between TB and altitude, precipitation, population density, and urbanisation.

Bias may have been introduced by the temporal mismatch between the TBPS data (2012–2019) and the climate data (1970–2000). While better matching data exist with similar geospatial resolution,^48^^,^^49^ these are not based on interpolated observations from weather stations like the WorldClim-2 data we used, but on reanalysis outputs from weather forecasting models which introduces other bias and uncertainty. Although the time-aggregated climate data period was long enough to capture temporal variation due to the El Niño Southern Oscillation, the TB surveys may have been done in outlier years. None collected data during or shortly after the 2014–16 El Niño event that caused extreme droughts Eastern and Southern Africa.^50^ La Niña was notable in 2016–17 with increased rainfall in Southern Africa, albeit marginal, which may have resulted in some bias in the observed association with rainfall and temperature for the South African survey (2017).^51^ Global warming may have affected weather conditions since 2000, which our data failed to capture, but the effect scale of the temporal mismatch was relatively small and changes over time would having occurred largely similarly across the geographies covered by our analyses.

Despite the survey samples being representative of the countries' populations, low participation rates among certain subgroups (e.g., young men) could have led to underestimation or overestimation of the observed associations. The nationally designed TB prevalence surveys used in this study were not originally intended for cross-country aggregation, which may have introduced inconsistencies. By design, selected clusters were predominantly located in the most densely populated areas of each country. In settings with high variation in population density, such as Nigeria and Sudan, this may have resulted in poor representation of certain regions, including those at different latitudes and with distinct environmental characteristics. Additionally, the distinction between rural and urban clusters can be subjective and context-dependent; a rural district with a high population density in one country may resemble an urban cluster in a country with an overall lower population density. By adjusting for both population density and urbanisation, we partially accounted for this variability. Finally, several ecological exposures were measured at the district level, therefore ecological fallacy — where the observed relationship between aggregated variables may differ from the relationship on the individual level — remains a limitation.^52^

It is crucial to recognise that the observed associations reflect a combination of TB incidence and duration of disease. They may therefore be modified by variation in access to diagnosis and treatment across districts. To delineate the interactions between TB incidence, undetected or untreated cases, and environmental-demographic determinants, a comparison between TB notifications and prevalence rates is necessary. This comparison would require improved data systems, conducting more regular surveys, and improving case detection, especially for individuals with asymptomatic TB.

In conclusion, in African countries with a high TB burden, TB prevalence appears to be linked to both geographic determinants, in particular altitude, latitude, precipitation, and temperature, and sociodemographic determinants, in particular population density and urbanisation. These data suggest that in addition to health system factors such as access to care, some of the variation in TB prevalence could be explained by environmental-demographic factors that merit further investigation.

## Contributors

TMIH, LS and FC conceptualized the study with methodological input by EK, IL, and ET who also assisted in contacting the investigators of the included surveys and the data curation. TMIH with assistance of DB and LS performed the formal analyses and visualized the data. TMIH wrote the original draft of the manuscript. EABA, NK, PK-C, BK, LBM-M, SM, JO, and ER were investigators for the surveys for which IPD were obtained and provided details on the data, their interpretation and the appropriate standardization of screening and diagnostic data. All authors provided comments on one or more draft versions and read and approved the final manuscript. TMIH, BD, LS and FC had access to all the data. Data were verified by TMIH, DB and LS.

## Data sharing statement

The study did not produce original data. De-identified individual participant data and a corresponding data dictionary defining each field in the set will be made available to others, conditional on approval by the providers of the respective original data. Additional related documents for the original datasets (e.g., study protocol, statistical analysis plan, and informed consent form) can be made available upon request by the providers of the respective original data. Criteria for sharing depend on the data providers' institutional and national regulations. The corresponding author can be contacted to help to initiate these processes.

All other data used in this study are from publicly available sources that are listed in Table 1 and are cited in the references.

## Declaration of interests

The authors declare no competing interests.

## References

[1] World Health Organization (2024).

[2] Cohen A., Mathiasen V.D., Schön T., Wejse C. (2019). The global prevalence of latent tuberculosis: a systematic review and meta-analysis. Eur Respir J.

[3] Harries A.D., Lawn S.D., Getahun H., Zachariah R., Havlir D.V. (2012). HIV and tuberculosis--science and implementation to turn the tide and reduce deaths. J Int AIDS Soc.

[4] Patra J., Jha P., Rehm J., Suraweera W. (2014). Tobacco smoking, alcohol drinking, diabetes, low body mass index and the risk of self-reported symptoms of active tuberculosis: individual participant data (IPD) meta-analyses of 72,684 individuals in 14 high tuberculosis burden countries. PLoS One.

[5] Narasimhan P., Wood J., Macintyre C.R., Mathai D. (2013). Risk factors for tuberculosis. Pulm Med.

[6] Santos L.G., Pires G.N., Azeredo Bittencourt L.R., Tufik S., Andersen M.L. (2012). Chronobiology: relevance for tuberculosis. Tuberculosis (Edinb).

[7] Gelaw Y.A., Yu W., Magalhães R.J.S., Assefa Y., Williams G. (2019). Effect of temperature and altitude difference on tuberculosis notification: a systematic review. J Glob Infect Dis.

[8] Ortblad K.F., Salomon J.A., Bärnighausen T., Atun R. (2015). Stopping tuberculosis: a biosocial model for sustainable development. Lancet.

[9] Popovic I., Soares Magalhaes R.J., Ge E. (2019). A systematic literature review and critical appraisal of epidemiological studies on outdoor air pollution and tuberculosis outcomes. Environ Res.

[10] Qin T., Hao Y., Wu Y. (2022). Association between averaged meteorological factors and tuberculosis risk: a systematic review and meta-analysis. Environ Res.

[11] Hargreaves J.R., Boccia D., Evans C.A., Adato M., Petticrew M., Porter J.D.H. (2011). The social determinants of tuberculosis: from evidence to action. Am J Public Health.

[12] Thorpe L.E., Frieden T.R., Laserson K.F., Wells C., Khatri G.R. (2004). Seasonality of tuberculosis in India: is it real and what does it tell us?. Lancet.

[13] Balcells M.E., Cerda J., Concha S. (2017). Regional solar radiation is inversely correlated with incidence and severity of tuberculosis in Chile. Epidemiol Infect.

[14] Im C., Kim Y. (2021). Spatial pattern of tuberculosis (TB) and related socio-environmental factors in South Korea, 2008-2016. PLoS One.

[15] Li Z., Liu Q., Chen L. (2024). Ambient air pollution contributed to pulmonary tuberculosis in China. Emerg Microbes Infect.

[16] Mohidem N., Osman M., Hashim Z., Muharam F.M., Mohd Elias S., Shaharudin R. (2021). Association of sociodemographic and environmental factors with spatial distribution of tuberculosis cases in Gombak, Selangor, Malaysia. PLoS One.

[17] Liyew A.M., Gebreyohannes E.A., Python A. (2025). Mapping tuberculosis prevalence in Africa using a Bayesian geospatial analysis. Commun Med.

[18] Xiang K., Xu Z., Hu Y.-Q. (2021). Association between ambient air pollution and tuberculosis risk: a systematic review and meta-analysis. Chemosphere.

[19] Boere T.M., Visser D.H., van Furth A.M., Lips P., Cobelens F.G.J. (2017). Solar ultraviolet B exposure and global variation in tuberculosis incidence: an ecological analysis. Eur Respir J.

[20] Li H., Ge M., Zhang M. (2022). Spatio-temporal distribution of tuberculosis and the effects of environmental factors in China. BMC Infect Dis.

[21] Li X.-X., Wang L.X., Zhang J. (2014). Exploration of ecological factors related to the spatial heterogeneity of tuberculosis prevalence in P. R. China. Glob Health Action.

[22] Sun W., Gong J., Zhou J. (2015). A spatial, social and environmental study of tuberculosis in China using statistical and GIS technology. Int J Environ Res Public Health.

[23] Zhang Q., Song W., Liu S. (2022). An ecological study of tuberculosis incidence in China, from 2002 to 2018. Front Public Health.

[24] Alene K.A., Python A., Weiss D.J. (2023). Mapping tuberculosis prevalence in Ethiopia using geospatial meta-analysis. Int J Epidemiol.

[25] Sadeq M., Bourkadi J.E. (2018). Spatiotemporal distribution and predictors of tuberculosis incidence in Morocco. Infect Dis Poverty.

[26] World Health Organization Tuberculosis prevalence surveys: a handbook 2011. https://iris.who.int/handle/10665/44481.

[27] Stuck L., Klinkenberg E., Abdelgadir Ali N. (2024). Prevalence of subclinical pulmonary tuberculosis in adults in community settings: an individual participant data meta-analysis. Lancet Infect Dis.

[28] GADM DoGAA (2022). Download GADM data (version 4.0). https://gadm.org/download_country.html.

[29] Affairs. UNOftCoH (2021). Ghana - subnational administrative boundaries. https://data.humdata.org/dataset/cod-ab-gha.

[30] Affairs UNOftCoH (2019). Lesotho - subnational administrative boundaries. https://data.humdata.org/dataset/cod-ab-lso.

[31] Affairs. UNOftCoH (2020). Uganda - subnational administrative boundaries. https://data.humdata.org/dataset/cod-ab-uga.

[32] van Donkelaar A., Hammer M.S., Bindle L. (2022). (SEDAC) NSDaAC.

[33] WorldPop (2022). Population density. http://www.worldpop.org.

[34] Fick S.E., Hijmans R.J. (2017). WorldClim 2: new 1-km spatial resolution climate surfaces for global land areas. Int J Climatol.

[35] Lab ADotGD Area database, version v4.4.1. https://globaldatalab.org/iwi/.

[36] Smits J., Steendijk R. (2015). The international wealth index (IWI). Soc Indic Res.

[37] Bates D., Mächler M., Bolker B., Walker S. (2015). Fitting linear mixed-effects models using lme 4. J Stat Software.

[38] World Health Organization WHO global air quality guidelines: particulate matter (PM2.5 and PM10), ozone, nitrogen dioxide, sulfur dioxide and carbon monoxide 2021. https://www.who.int/publications/i/item/9789240034228.

[39] Vargas M.H., Furuya M.E.Y., Pérez-Guzmán C. (2004). Effect of altitude on the frequency of pulmonary tuberculosis. Int J Tuberc Lung Dis.

[40] World Bank Group Average precipitation in depth (mm per year). https://data.worldbank.org/indicator/AG.LND.PRCP.MM?most_recent_value_desc=true.

[41] Ralph A.P., Lucas R.M., Norval M. (2013). Vitamin D and solar ultraviolet radiation in the risk and treatment of tuberculosis. Lancet Infect Dis.

[42] de Abreu E Silva M., Di Lorenzo Oliveira C., Teixeira Neto R.G., Camargos P.A. (2016). Spatial distribution of tuberculosis from 2002 to 2012 in a midsize city in Brazil. BMC Public Health.

[43] Selvaraju S., Velayutham B., Rao R. (2023). Prevalence and factors associated with tuberculosis infection in India. J Infect Public Health.

[44] Vlahov D., Freudenberg N., Proietti F. (2007). Urban as a determinant of health. J Urban Health.

[45] United Nations Human Settlements Programme (UN-HABITAT) (2022). Population living in slums (% of the urban population) - Ghana, Lesotho, Nigeria, Uganda, Zambia, Sudan, South Africa. https://data.worldbank.org/indicator/EN.POP.SLUM.UR.ZS?end=2018&locations=GH-LS-NG-UG-ZM-SD-ZA&start=2018&view=bar.

[46] Maulide Cane R., Melesse D.Y., Kayeyi N. (2021). HIV trends and disparities by gender and urban-rural residence among adolescents in Sub-Saharan Africa. Reprod Health.

[47] Lönnroth K., Holtz T.H., Cobelens F. (2009). Inclusion of information on risk factors, socio-economic status and health seeking in a tuberculosis prevalence survey. Int J Tuberc Lung Dis.

[48] (C3S) CCCS (2019). Climate Data Store (CDS).

[49] Karger D.N., Schmatz D.R., Dettling G., Zimmermann N.E. (2020). High-resolution monthly precipitation and temperature time series from 2006 to 2100. Sci Data.

[50] Kolusu S.R., Shamsudduha M., Todd M.C. (2019). The El Niño event of 2015–2016: climate anomalies and their impact on groundwater resources in East and Southern Africa. Hydrol Earth Syst Sci.

[51] Wang B., Sun W., Jin C. (2023). Understanding the recent increase in multiyear La Niñas. Nat Clim Chang.

[52] Greenland S. (2001). Ecologic versus individual-level sources of bias in ecologic estimates of contextual health effects. Int J Epidemiol.

