# Supplementary material for: Environmental-demographic determinants associated with tuberculosis prevalence in seven African countries: an aggregated dataset analysis
Source: eClinicalMedicine. 2026 Jan 28;92:103773. doi: 10.1016/j.eclinm.2026.103773 (PMC12874274; doi:10.1016/j.eclinm.2026.103773)
Supplement: Supplementary Tables and Figures [file mmc1.docx]

**Supplementary appendix 1**

Supplementary to: Environmental-demographic determinants associated with tuberculosis prevalence in seven African countries.

**Table of contents**

[Supplementary Table S1: Summary of design, methods, and results of National Tuberculosis Prevalence Surveys in Ghana, Lesotho, Nigeria, South Africa, Sudan, Uganda, and Zambia 3](#_Toc211503934)

[​Supplementary Table S2: Summary of deterministic data linkage and participants exclusions 4](#_Toc211503935)

[Supplementary Equation E1: General Linear Mixed Models in matrix notation 5](#_Toc211503936)

[Supplementary Table S3: Overview of Covariate Variance Inflation (VIF) Interpretation 6](#_Toc211503938)

[Supplementary Table S3.A: Sensitivity analyses omitting latitude: odds ratio and 95% confidence interval for tuberculosis prevalence in 322 615 individuals in Ghana, Lesotho, Nigeria, Sudan, South-Africa, Uganda, and Zambia 7](#_Toc211503953)

[Supplementary Table S3.B: Sensitivity analyses omitting PM_2∙5_: odds ratio and 95% confidence interval for tuberculosis prevalence in 322 615 individuals in Ghana, Lesotho, Nigeria, Sudan, South-Africa, Uganda, and Zambia 8](#_Toc211503954)

[Supplementary Table S4: Interaction urbanisation and population density: odds ratio and 95% confidence interval for tuberculosis prevalence in 322 615 individuals in Ghana, Lesotho, Nigeria, Sudan, South-Africa, Uganda, and Zambia 9](#_Toc211503955)

[Supplementary Table S5: Interaction altitude and sex: odds ratio and 95% confidence interval for tuberculosis prevalence in 322 615 individuals in Ghana, Lesotho, Nigeria, Sudan, South-Africa, Uganda, and Zambia 10](#_Toc211503956)

[Supplementary Table S6: Interaction latitude and sex: odds ratio and 95% confidence interval for tuberculosis prevalence in 322 615 individuals in Ghana, Lesotho, Nigeria, Sudan, South-Africa, Uganda, and Zambia 11](#_Toc211503957)

[Supplementary Table S7: Interaction precipitation and sex: odds ratio and 95% confidence interval for tuberculosis prevalence in 322 615 individuals in Ghana, Lesotho, Nigeria, Sudan, South-Africa, Uganda, and Zambia 12](#_Toc211503958)

[Supplementary Table S8: Interaction temperature and latitude: odds ratio and 95% confidence interval for tuberculosis prevalence in 322 615 individuals in Ghana, Lesotho, Nigeria, Sudan, South-Africa, Uganda, and Zambia 13](#_Toc211503959)

[Supplementary Figure S1: Visualisation of the distribution of prevalence of tuberculosis 14](#_Toc211503960)

[Supplementary Figure S2: Correlation Matrix 15](#_Toc211503961)

[References Supplementary Material 16](#_Toc211503962)

# **Supplementary Table S1: Summary of design, methods, and results of National Tuberculosis Prevalence Surveys in Ghana, Lesotho, Nigeria, South Africa, Sudan, Uganda, and Zambia**

|  | Ghana^1^ | Lesotho^2^ | Nigeria^3^ | South Africa^4^ | Sudan^5^ | Uganda^6^ | Zambia^7^ |
| --- | --- | --- | --- | --- | --- | --- | --- |
| Year | 2013 | 2019 | 2012 | 2017 | 2013—14 | 2014—15 | 2013—14 |
| Total population in the year of the survey (n)^8^ | 26 607 641 | 2 125 641 | 167 228 803 | 53 873 616 | 40 679 828 | 36 911 530 | 15 399 793 |
| Participants screened (n) | 67 740 | 26 857 | 41 363 | 32 222 | 81 674 | 86 108 | 46 099 |
| Presumptive TB screening | | | | | | | |
| Definition screen positive | Cough ≥ 2 weeks;  abnormal shadows on CXR | Cough of any duration/fever/ weight loss/night sweats; any CXR abnormality | Cough ≥ 2 weeks;  abnormal shadows on CXR | Cough of any duration/fever/ weight loss/night sweats; any CXR abnormality | Cough ≥ 2 weeks; currently on TB treatment;  abnormal shadows on CXR | Cough ≥ 2 weeks;  abnormal shadows on CXR; did not take CXR | Cough/fever/chest pain ≥ 2 weeks; abnormal shadows on CXR |
| Diagnostic tests | | | | | | | |
| Smear examination | 2 ZN* | No | 2 ZN | No | 2 FM | 2 ZN | 2 ZN |
| Culture | 2 MGIT | MGIT | 2 LJ | MGIT | 2 Ogawa | 2 LJ | 2 MGIT |
| Xpert MTB/RIF or Xpert Ultra | Smear-positive or contaminated cultures | All screen positives | No | All screen positives | No | Smear-positive or contaminated cultures | Smear-positive or contaminated cultures |
| Bacteriologically confirmed pulmonary TB | | | | | | | |
| Number of cases (n) | 202 | 132 | 144 | 234 | 112 | 160 | 265 |
| Prevalence per 100,000 population aged ≥15 years | 356 | 581 | 524 | 852 | 183 | 401 | 638 |
| Further examination | | | | | | | |
| In-depth interview | No | No | No | No | Yes | No | Yes |

*Abbreviations used: TB = tuberculosis, CXR = Chest x-ray, ZN = Ziehl-Neelsen stain, FM = fluorescent microscopy, MGIT = Mycobacterial growth indicator tube, LJ = Löwenstein-Jensen, MTB = Mycobacterium Tuberculosis; RIF = Rifampicin.*

** In Ghana, Ziehl-Neelsen smears used the concentrated method.*

# ​**Supplementary Table S2: Summary of deterministic data linkage and participants exclusions**

| **Country** | **Administrative level used for deterministic data linkage** | **Districts available in GADM/HDX database covered by IPD (n)** | **Districts available in GADM/HDX database not covered by IPD (n)** | **Total population screened who did not receive previous TB treatment** | **Participants excluded whose administrative IPD could not be linked to GADM/HDX database (n)** | **Total population included in analysis** |
| --- | --- | --- | --- | --- | --- | --- |
| Ghana | 2 | 91 | 169 | 61 367 | 0 | 61 367 |
| Lesotho | 2 | 42 | 36 | 19 541 | 913  (empty (59),  number instead of name (522),  no match based on spelling (322),  district with 1 person (10)) | 18 638 |
| Nigeria | 2 | 70 | 0 | 43 584 | 0 | 43 584 |
| South Africa | 2 | 61 | 43 | 32 222 | 0 | 32 222 |
| Sudan | 1 | 30 | 3 | 81 674 | 0 | 81 674 |
| Uganda | 2 | 57 | 78 | 40 286 | 0 | 40 286 |
| Zambia | 2 | 49 | 23 | 44 854 | 0 | 44 854 |
| Total |  | 400 |  | 323 528 | 913 | 322 615 |

*Abbreviations used: GADM = Database of Global Administrative Areas, HDX = Humanitarian Data Exchange, IPD= Individual Participant Data.*

# **Supplementary Equation E1: General Linear Mixed Models in matrix notation**

The general form of the model is^9^

$$\boldsymbol{y=X\beta+Zu+\varepsilon}$$

where:

- *y* is the *n×1* vector of observed responses
- *X* is the *n×p* design matrix for fixed‐effect predictors
- *β* is the *p×1* vector of fixed‐effect coefficient.
- *Z* is the *n×q* design matrix for random‐effect predictors
- *u* is the *q×1* vector of random effects
- *ε* is the *n×1* vector of residual errors.

**Supplementary Table S3: Overview of Variance Inflation Factors (VIFs) for all covariates**

| **Covariate** | **Variance Inflation Factor (VIF)** | **Interpretation** |
| --- | --- | --- |
| Altitude (m) | 8∙59 | Moderate Correlation |
| Latitude (absolute degrees) | 11∙84 | High Correlation |
| Precipitation (mm) | 5∙81 | Moderate Correlation |
| PM_2∙5_ (*µg/m^3^*) | 10∙33 | High Correlation |
| Population density, logarithm of mean people per km² | 2∙20 | Low Correlation |
| Solar radiation (MJ/m^2^/day) | 3∙39 | Low Correlation |
| Temperature (degrees Celsius) | 6∙97 | Moderate Correlation |
| Urbanisation | 1∙52 | Low Correlation |
| International Wealth Index | 2∙88 | Low Correlation |
| Sex | 1∙02 | Low Correlation |
| Age | 1∙03 | Low Correlation |

*Abbreviations used: m = metres, mm = millimetres, (k)m^2^ = square (kilo)metre, PM2∙5 = particulate matter with aerodynamic diameter ≤2∙5 µm, µg/m^3^ = micrograms per cubic metre air.*

# **Supplementary Table S3.A: Sensitivity analyses omitting latitude: odds ratio and 95% confidence interval for tuberculosis prevalence in 322 615 individuals in Ghana, Lesotho, Nigeria, Sudan, South-Africa, Uganda, and Zambia**

|  | **Odds ratio (95% CI)** | **P-value** |
| --- | --- | --- |
| **Environmental-demographic variables** | | |
| Altitude (m) |  |  |
| *≤450 m* | ref |  |
| *>450, ≤900 m* | 0∙89 (0∙62—1∙28) | 0∙53 |
| >90*0 m* | 0∙53 (0∙33—0∙87) | 0∙01 |
| Precipitation (mm) |  |  |
| *>0, ≤50* | ref |  |
| *>50, ≤100* | 0∙67 (0∙50—0∙90) | <0∙01 |
| *>100* | 1∙07 (0∙74—1∙55) | 0∙72 |
| PM_2∙5_ (*µg/m^3^*) |  |  |
| *≤20* | ref |  |
| *>20, ≤40* | 1∙31 (0∙89—1∙91) | 0∙17 |
| *>40* | 0∙91 (0∙48—1∙70) | 0∙76 |
| Population density, logarithm of mean people per km² | 1∙04 (0∙99—1∙10) | 0∙13 |
| Solar radiation (MJ/m^2^/day) | 1∙16 (1∙06—1∙26) | <0∙01 |
| Temperature (degrees Celsius) | 0∙93 (0∙88—0∙98) | 0∙01 |
| Urbanisation |  |  |
| *Rural* | ref |  |
| *Urban* | 1∙29 (1∙09—1∙51) | <0∙01 |
| International Wealth Index | 1∙00 (1∙00—1∙01) | 0∙27 |
| **Individual variables** |  | |
| Sex |  |  |
| *Female* | ref |  |
| *Male* | 1∙83 (1∙60—2∙09) | <0∙01 |
| Age group (years) |  |  |
| *15—24* | ref |  |
| *25—34* | 1∙58 (1∙26—1∙99) | <0∙01 |
| *35—44* | 1∙48 (1∙17—1∙87) | <0∙01 |
| *45—54* | 1∙19 (0∙93—1∙52) | 0∙18 |
| *55—64* | 1∙08 (0∙84—1∙40) | 0∙55 |
| *65—74* | 1∙01 (0∙76—1∙35) | 0∙92 |
| *≥75* | 1∙05 (0∙78—1∙41) | 0∙77 |

*Abbreviations used: CI95% = 9*5% confidence interval, *m = metres, ref = reference category, mm = millimetres, PM_2∙5_ = particulate matter with aerodynamic diameter ≤2∙5 µm, µg/m^3^ = micrograms per cubic metre air, km^2^ = square kilometre, MJ/m^2^/day = Megajoule per square metre per day, sd = standard deviation.*

# **Supplementary Table S3.B: Sensitivity analyses omitting PM_2_**_∙_**_5_: odds ratio and 95% confidence interval for tuberculosis prevalence in 322 615 individuals in Ghana, Lesotho, Nigeria, Sudan, South-Africa, Uganda, and Zambia**

|  | **Odds ratio (95% CI)** | **P-value** |
| --- | --- | --- |
| **Environmental-demographic variables** | | |
| Altitude (m) |  |  |
| *≤450 m* | ref |  |
| *>450, ≤900 m* | 0∙79 (0∙56—1∙13) | 0∙20 |
| >90*0 m* | 0∙52 (0∙32—0∙84) | <0∙01 |
| Latitude (absolute degrees) |  |  |
| *≤7*∙6° | ref |  |
| *>7*∙6, *≤14*∙6° | 2∙21 (1∙60—3∙06) | <0∙01 |
| *>14*∙6° | 1∙67 (1∙03—2∙73) | 0∙04 |
| Precipitation (mm) |  |  |
| *>0, ≤50* | ref |  |
| *>50, ≤100* | 0∙62 (0∙46—0∙84) | <0∙01 |
| *>100* | 1∙01 (0∙71—1∙44) | 0∙96 |
| Population density, logarithm of mean people per km² | 1∙06 (1∙00—1∙13) | 0∙02 |
| Solar radiation (MJ/m^2^/day) | 1∙03 (0∙94—1∙26) | 0∙52 |
| Temperature (degrees Celsius) | 0∙93 (0∙88—0∙98) | 0∙01 |
| Urbanisation |  |  |
| *Rural* | ref |  |
| *Urban* | 1∙31 (1∙12—1∙54) | <0∙01 |
| International Wealth Index | 1∙00 (1∙00—1∙01) | 0∙13 |
| **Individual variables** |  | |
| Sex |  |  |
| *Female* | ref |  |
| *Male* | 1∙83 (1∙60—2∙09) | <0∙01 |
| Age group (years) |  |  |
| *15—24* | ref |  |
| *25—34* | 1∙59 (1∙26—1∙99) | <0∙01 |
| *35—44* | 1∙48 (1∙17—1∙86) | <0∙01 |
| *45—54* | 1∙18 (0∙92—1∙51) | 0∙19 |
| *55—64* | 1∙06 (0∙82—1∙38) | 0∙64 |
| *65—74* | 1∙01 (0∙76—1∙34) | 0∙97 |
| *≥75* | 1∙05 (0∙78—1∙41) | 0∙75 |

*Abbreviations used: CI95% = 9*5% confidence interval, *m = metres, ref = reference category, mm = millimetres, PM_2∙5_ = particulate matter with aerodynamic diameter ≤2∙5 µm, µg/m^3^ = micrograms per cubic metre air, km^2^ = square kilometre, MJ/m^2^/day = Megajoule per square metre per day, sd = standard deviation.*

**Supplementary Table S4: Interaction urbanisation and population density: odds ratio and 95% confidence interval for tuberculosis prevalence in 322 615 individuals in Ghana, Lesotho, Nigeria, Sudan, South-Africa, Uganda, and Zambia**

|  | **Odds ratio (95% CI)** | **P-value** |
| --- | --- | --- |
| **Environmental-demographic variables** | | |
| Altitude (m) |  |  |
| *≤450 m* | ref |  |
| *>450, ≤900 m* | 0∙77 (0∙54—1∙10) | 0∙15 |
| >90*0 m* | 0∙52 (0∙32—0∙84) | 0∙01 |
| Latitude (absolute degrees) |  |  |
| *≤7*∙6° | ref |  |
| *>7*∙6, *≤14*∙6° | 2∙07 (1∙48—2∙90) | <0∙001 |
| *>14*∙6° | 1∙50 (0∙90—2∙48) | 0∙14 |
| Precipitation (mm) |  |  |
| *>0, ≤50* | refref |  |
| *>50, ≤100* | 0∙61 (0∙45—0∙83) | 0∙002 |
| *>100* | 0∙92 (0∙63—1∙34) | 0∙67 |
| PM_2∙5_ (*µg/m^3^*) |  |  |
| *≤20* | ref |  |
| *>20, ≤40* | 1∙07 (0∙72—1∙60) | 0∙73 |
| *>40* | 0∙90 (0∙44—1∙53) | 0∙75 |
| Population density, logarithm of mean people per km² | 1∙13 (1∙04—1∙25) | 0∙01 |
| Solar radiation (MJ/m^2^/day) | 1∙02 (0∙93—1∙14) | 0∙59 |
| Temperature (degrees Celsius) | 0∙93 (0∙88—0∙98) | 0∙01 |
| Urbanisation |  |  |
| *Rural* | ref |  |
| *Urban* | 1∙96 (1∙20—3∙21) | 0∙001 |
| International Wealth Index | 1∙01 (1∙00—1∙01) | 0∙16 |
| **Individual variables** |  | |
| Sex |  |  |
| *Female* | ref |  |
| *Male* | 1∙83 (1∙60—2∙09) | <0∙001 |
| Age group (years) |  |  |
| *15—24* | ref |  |
| *25—34* | 1∙58 (1∙26—1∙99) | <0∙001 |
| *35—44* | 1∙48 (1∙17—1∙87) | 0∙001 |
| *45—54* | 1∙19 (0∙93—1∙52) | 0∙18 |
| *55—64* | 1∙07 (0∙83—1∙39) | 0∙59 |
| *65—74* | 1∙01 (0∙76—1∙35) | 0∙93 |
| *≥75* | 1∙05 (0∙78—1∙42) | 0∙74 |
| **Interaction term: Population density * Urbanisation** | | |
| *Population density * Rural* | ref |  |
| *Population density * Urban* | 0∙92 (0∙84—1∙01) | 0∙09 |

*Abbreviations used: CI95% = 9*5% confidence interval, *m = metres, ref = reference category, mm = millimetres, PM_2∙5_ = particulate matter with aerodynamic diameter ≤2∙5 µm, µg/m^3^ = micrograms per cubic metre air, km^2^ = square kilometre, MJ/m^2^/day = Megajoule per square metre per day, sd = standard deviation.*

# **Supplementary Table S5: Interaction altitude and sex: odds ratio and 95% confidence interval for tuberculosis prevalence in 322 615 individuals in Ghana, Lesotho, Nigeria, Sudan, South-Africa, Uganda, and Zambia**

|  | **Odds ratio (95% CI)** | **P-value** |
| --- | --- | --- |
| **Environmental-demographic variables** | | |
| Altitude (m) |  |  |
| *≤450 m* | ref |  |
| *>450, ≤900 m* | 0∙75 (0∙54—1∙10) | 0∙15 |
| >90*0 m* | 0∙56 (0∙32—0∙84) | 0∙01 |
| Latitude (absolute degrees) |  |  |
| *≤7*∙6° | ref |  |
| *>7*∙6, *≤14*∙6° | 2∙07 (1∙48—2∙90) | <0∙001 |
| *>14*∙6° | 1∙49 (0∙90—2∙48) | 0∙14 |
| Precipitation (mm) |  |  |
| *>0, ≤50* | ref |  |
| *>50, ≤100* | 0∙63 (0∙45—0∙83) | 0∙002 |
| *>100* | 0∙96 (0∙63—1∙34) | 0∙67 |
| PM_2∙5_ (*µg/m^3^*) |  |  |
| *≤20* | ref |  |
| *>20, ≤40* | 1∙04 (0∙72—1∙60) | 0∙73 |
| *>40* | 0∙82 (0∙44—1∙53) | 0∙75 |
| Population density, logarithm of mean people per km² | 1∙07 (1∙04—1∙25) | 0∙01 |
| Solar radiation (MJ/m^2^/day) | 1∙03 (0∙93—1∙14) | 0∙59 |
| Temperature (degrees Celsius) | 0∙93 (0∙88—0∙98) | 0∙01 |
| Urbanisation |  |  |
| *Rural* | ref |  |
| *Urban* | 1∙31 (1∙20—3∙21) | 0∙001 |
| International Wealth Index | 1∙01 (1∙00—1∙01) | 0∙16 |
| **Individual variables** |  | |
| Sex |  |  |
| *Female* | ref |  |
| *Male* | 1∙93 (1∙60—2∙09) | <0∙001 |
| Age group (years) |  |  |
| *15—24* | ref |  |
| *25—34* | 1∙59 (1∙26—1∙99) | <0∙001 |
| *35—44* | 1∙48 (1∙17—1∙87) | 0∙001 |
| *45—54* | 1∙19 (0∙93—1∙52) | 0∙18 |
| *55—64* | 1∙07 (0∙83—1∙39) | 0∙59 |
| *65—74* | 1∙01 (0∙76—1∙35) | 0∙93 |
| *≥75* | 1∙05 (0∙78—1∙42) | 0∙74 |
| **Interaction term: Altitude (m) * Sex** | | |
| *≤450 m* ** Male* | ref |  |
| *>450, ≤900 m* ** Male* | 1∙09 (0∙72—1∙65) | 0∙68 |
| *>900 m* ** Male* | 0∙88 (0∙65—1∙18) | 0∙38 |

*Abbreviations used: CI95% = 9*5% confidence interval, *m = metres, ref = reference category, mm = millimetres, PM_2∙5_ = particulate matter with aerodynamic diameter ≤2∙5 µm, µg/m^3^ = micrograms per cubic metre air, km^2^ = square kilometre, MJ/m^2^/day = Megajoule per square metre per day, sd = standard deviation.*

# **Supplementary Table S6: Interaction latitude and sex: odds ratio and 95% confidence interval for tuberculosis prevalence in 322 615 individuals in Ghana, Lesotho, Nigeria, Sudan, South-Africa, Uganda, and Zambia**

|  | **Odds ratio (95% CI)** | **P-value** |
| --- | --- | --- |
| **Environmental-demographic variables** | | |
| Altitude (m) |  |  |
| *≤450 m* | ref |  |
| *>450, ≤900 m* | 0∙81 (0∙56—1∙15) | 0∙23 |
| >90*0 m* | 0∙53 (0∙32—0∙86) | 0∙01 |
| Latitude (absolute degrees) |  |  |
| *≤7*∙6° | ref |  |
| *>7*∙6, *≤14*∙6° | 2∙76 (1∙89—4∙03) | <0∙001 |
| *>14*∙6° | 1∙82 (1∙06—3∙11) | 0∙03 |
| Precipitation (mm) |  |  |
| *>0, ≤50* | ref |  |
| *>50, ≤100* | 0∙62 (0∙46—0∙83) | 0∙002 |
| *>100* | 0∙94 (0∙65—1∙37) | 0∙76 |
| PM_2∙5_ (*µg/m^3^*) |  |  |
| *≤20* | ref |  |
| *>20, ≤40* | 1∙03 (0∙69—1∙52) | 0∙89 |
| *>40* | 0∙80 (0∙43—1∙49) | 0∙48 |
| Population density, logarithm of mean people per km² | 1∙07 (1∙04—1∙25) | 0∙02 |
| Solar radiation (MJ/m^2^/day) | 1∙04 (0∙94—1∙15) | 0∙46 |
| Temperature (degrees Celsius) | 0∙93 (0∙88—0∙99) | 0∙01 |
| Urbanisation |  |  |
| *Rural* | ref |  |
| *Urban* | 1∙31 (1∙11—1∙54) | 0∙001 |
| International Wealth Index | 1∙01 (1∙00—1∙02) | 0∙09 |
| **Individual variables** |  | |
| Sex |  |  |
| *Female* | ref |  |
| *Male* | 2∙58 (2∙01—3∙31) | <0∙001 |
| Age group (years) |  |  |
| *15—24* | ref |  |
| *25—34* | 1∙58 (1∙26—1∙99) | <0∙001 |
| *35—44* | 1∙47 (1∙17—1∙86) | 0∙001 |
| *45—54* | 1∙19 (0∙93—1∙52) | 0∙17 |
| *55—64* | 1∙07 (0∙83—1∙39) | 0∙59 |
| *65—74* | 1∙01 (0∙76—1∙35) | 0∙92 |
| *≥75* | 1∙06 (0∙79—1∙43) | 0∙74 |
| **Interaction term: Latitude (absolute degrees) * Sex** | | |
| *≤7*∙6° ** Male* | ref |  |
| *>7*∙6, *≤14*∙6° ** Male* | 0∙57 (0∙41—0∙80) | <0∙001 |
| *> 14*∙6° ** Male* | 0∙66 (0∙47—0∙92) | 0∙01 |

*Abbreviations used: CI95% = 9*5% confidence interval, *m = metres, ref = reference category, mm = millimetres, PM_2∙5_ = particulate matter with aerodynamic diameter ≤2∙5 µm, µg/m^3^ = micrograms per cubic metre air, km^2^ = square kilometre, MJ/m^2^/day = Megajoule per square metre per day, sd = standard deviation.*

# **Supplementary Table S7: Interaction precipitation and sex: odds ratio and 95% confidence interval for tuberculosis prevalence in 322 615 individuals in Ghana, Lesotho, Nigeria, Sudan, South-Africa, Uganda, and Zambia**

|  | **Odds ratio (95% CI)** | **P-value** |
| --- | --- | --- |
| **Environmental-demographic variables** | | |
| Altitude (m) |  |  |
| *≤450 m* | ref |  |
| *>450, ≤900 m* | 0∙80 (0∙56—1∙14) | 0∙21 |
| >90*0 m* | 0∙52 (0∙32—0∙84) | 0∙01 |
| Latitude (absolute degrees) |  |  |
| *≤7*∙6° | ref |  |
| *>7*∙6, *≤14*∙6° | 2∙05 (1∙46—2∙86) | <0∙001 |
| *>14*∙6° | 1∙48 (0∙90—2∙44) | 0∙12 |
| Precipitation (mm) |  |  |
| *>0, ≤50* | ref |  |
| *>50, ≤100* | 0∙67 (0∙47—0∙95) | 0∙003 |
| *>100* | 0∙89 (0∙58—1∙37) | 0∙61 |
| PM_2∙5_ (*µg/m^3^*) |  |  |
| *≤20* | ref |  |
| *>20, ≤40* | 1∙04 (0∙70—1∙54) | 0∙84 |
| *>40* | 0∙82 (0∙44—1∙54) | 0∙54 |
| Population density, logarithm of mean people per km² | 1∙07 (1∙01—1∙13) | 0∙02 |
| Solar radiation (MJ/m^2^/day) | 1∙04 (0∙94—1∙15) | 0∙48 |
| Temperature (degrees Celsius) | 0∙93 (0∙88—0∙98) | 0∙01 |
| Urbanisation |  |  |
| *Rural* | ref |  |
| *Urban* | 1∙31 (1∙11—1∙54) | 0∙001 |
| International Wealth Index | 1∙01 (1∙00—1∙02) | 0∙10 |
| **Individual variables** |  | |
| Sex |  |  |
| *Female* | ref |  |
| *Male* | 1∙88 (1∙41—2∙52) | <0∙001 |
| Age group (years) |  |  |
| *15—24* | ref |  |
| *25—34* | 1∙58 (1∙26—1∙99) | <0∙001 |
| *35—44* | 1∙48 (1∙17—1∙87) | 0∙001 |
| *45—54* | 1∙19 (0∙93—1∙52) | 0∙17 |
| *55—64* | 1∙07 (0∙83—1∙39) | 0∙59 |
| *65—74* | 1∙01 (0∙76—1∙35) | 0∙94 |
| *≥75* | 1∙05 (0∙79—1∙43) | 0∙74 |
| **Interaction term: Precipitation (mm) * Sex** | | |
| *>0, ≤50 * Male* | ref |  |
| *>50, ≤100 * Male* | 0∙87 (0∙61—1∙24) | 0∙43 |
| *>100 * Male* | 1∙09 (0∙76—1∙57) | 0∙64 |

*Abbreviations used: CI95% = 9*5% confidence interval, *m = metres, ref = reference category, mm = millimetres, PM_2∙5_ = particulate matter with aerodynamic diameter ≤2∙5 µm, µg/m^3^ = micrograms per cubic metre air, km^2^ = square kilometre, MJ/m^2^/day = Megajoule per square metre per day, sd = standard deviation.*

# **Supplementary Table S8: Interaction temperature and latitude: odds ratio and 95% confidence interval for tuberculosis prevalence in 322 615 individuals in Ghana, Lesotho, Nigeria, Sudan, South-Africa, Uganda, and Zambia**

|  | **Odds ratio (95% CI)** | **P-value** |
| --- | --- | --- |
| **Environmental-demographic variables** | | |
| Altitude (m) |  |  |
| *≤450 m* | ref |  |
| *>450, ≤900 m* | 0∙95 (0∙63—1∙41) | 0∙78 |
| >90*0 m* | 0∙65 (0∙38—1∙13) | 0∙13 |
| Latitude (absolute degrees) |  |  |
| *≤7*∙6° | ref |  |
| *>7*∙6, *≤14*∙6° | 0∙07 (0∙001—2∙63) | 0∙15 |
| *>14*∙6° | 0∙46 (0∙03—7∙31) | 0∙59 |
| Precipitation (mm) |  |  |
| *>0, ≤50* | ref |  |
| *>50, ≤100* | 0∙46 (0∙48—0∙88) | 0∙01 |
| *>100* | 1∙01 (0∙69—1∙47) | 0∙95 |
| PM2∙5 (*µg/m^3^*) |  |  |
| *≤20* | ref |  |
| *>20, ≤40* | 0∙94 (0∙62—1∙42) | 0∙76 |
| *>40* | 0∙86 (0∙46—1∙62) | 0∙64 |
| Population density, logarithm of mean people per km² | 1∙06 (1∙01—1∙13) | 0∙07 |
| Solar radiation (MJ/m^2^/day) | 1∙03 (0∙93—1∙14) | 0∙63 |
| Temperature (degrees Celsius) | 0∙90 (0∙83—0∙98) | 0∙02 |
| Urbanisation | = |  |
| *Rural* | ref |  |
| *Urban* | 1∙32 (1∙12—1∙56) | 0∙001 |
| International Wealth Index | 1∙01 (1∙00—1∙02) | 0∙03 |
| **Individual variables** |  | |
| Sex |  |  |
| *Female* | ref |  |
| *Male* | 1∙82 (1∙41—2∙52) | <0∙001 |
| Age group (years) |  |  |
| *15—24* | ref |  |
| *25—34* | 1∙59 (1∙26—1∙99) | <0∙001 |
| *35—44* | 1∙49 (1∙17—1∙87) | 0∙001 |
| *45—54* | 1∙20 (0∙93—1∙52) | 0∙15 |
| *55—64* | 1∙09 (0∙83—1∙39) | 0∙54 |
| *65—74* | 1∙02 (0∙76—1∙35) | 0∙88 |
| *≥75* | 1∙06 (0∙79—1∙43) | 0∙74 |
| **Interaction term: Latitude (absolute degrees) * Temperature (degrees Celsius)** | | |
| *≤7*∙6° ** Temperature* | ref |  |
| *>7*∙6, *≤14*∙6° ** Temperature* | 1∙13 (0∙99—1∙30) | 0∙07 |
| *> 14*∙6° ** Temperature* | 1∙02 (0∙91—1∙14) | 0∙73 |

*Abbreviations used: CI95% = 9*5% confidence interval, *m = metres, ref = reference category, mm = millimetres, PM_2∙5_ = particulate matter with aerodynamic diameter ≤2∙5 µm, µg/m^3^ = micrograms per cubic metre air, km^2^ = square kilometre, MJ/m^2^/day = Megajoule per square metre per day, sd = standard deviation.*


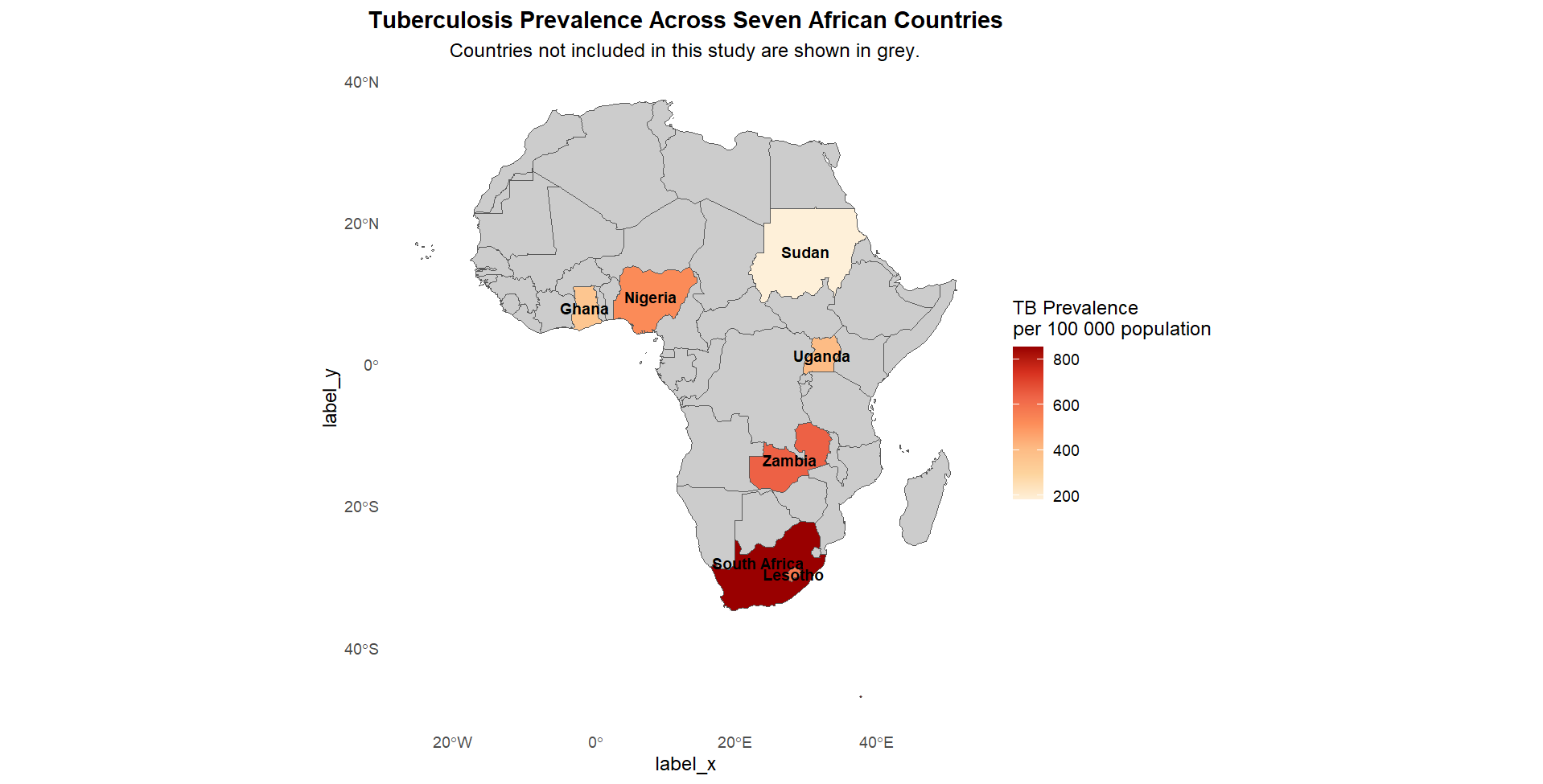


**Supplementary Figure S1: Visualisation of the distribution of prevalence of tuberculosis**

Coloured estimates are averages of tuberculosis prevalence as in country reports (Ghana (2013)^1^,

Lesotho (2019)^2^, Nigeria (2012)^3^, South Africa (2017)^4^, Sudan (2013-14)^5^, Uganda (2014-15)^6^, Zambia (2013-14)^7^)


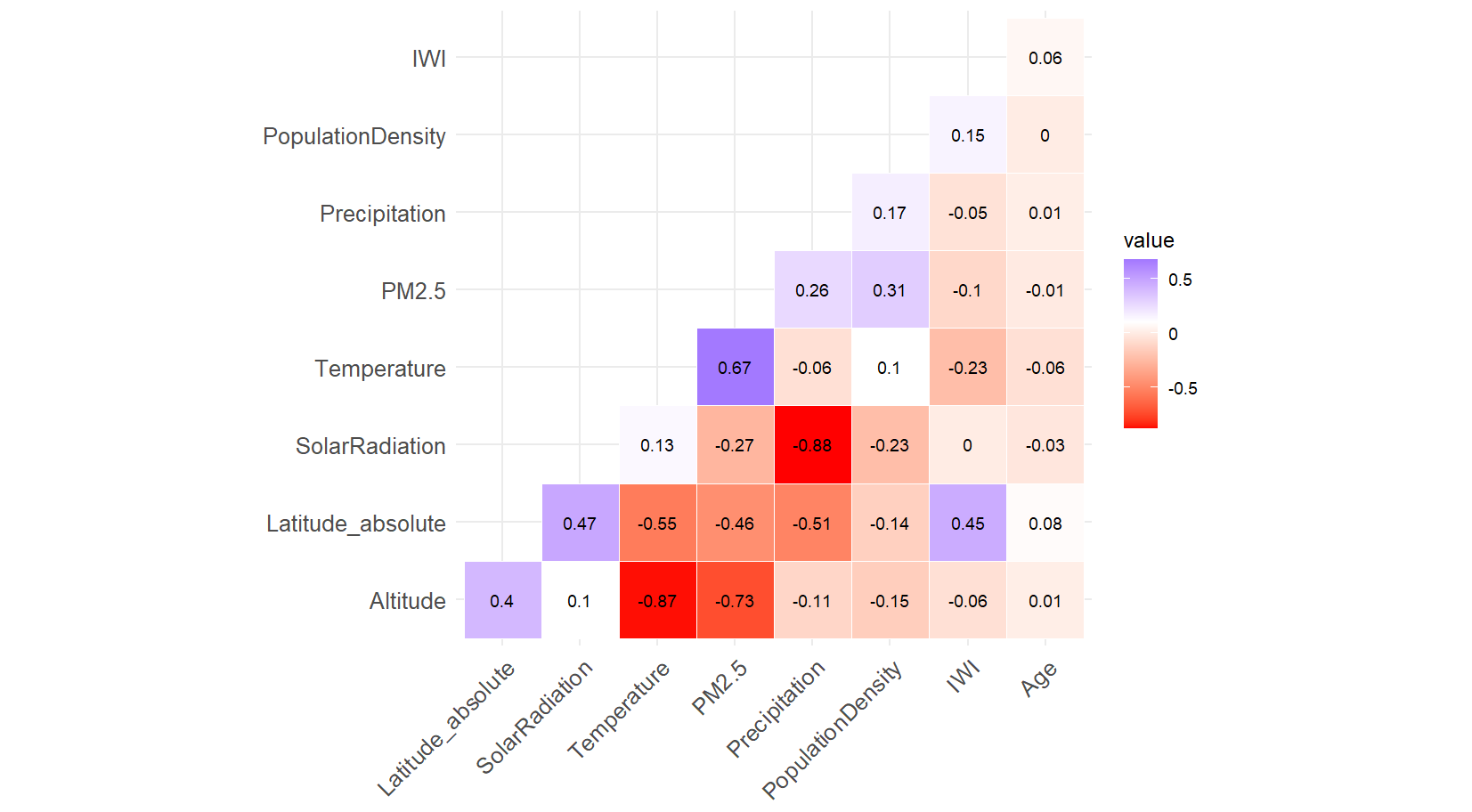


# **Supplementary Figure S2: Correlation Matrix**

Estimates are based on original variables (i.e. without application log transformation or categorisation to those variables that were not linear).

*Abbreviations used: IWI = International Wealth Index, PM_2∙5_ = particulate matter with aerodynamic diameter.*

# **References Supplementary Material**

1. Programme GHSNTC. Ghanaian National Population Based Tuberculosis Prevalence Survey in 2013. Accra, 2015.

2. Matji R ∙ Maama L ∙ Roscigno G ∙ et al. Policy and programmatic directions for the Lesotho tuberculosis programme: Findings of the national tuberculosis prevalence survey, 2019. *PLoS One* 2023; **18**(3): e0273245.

3. Health FMo. Report FIRST National TB Prevalence Survey 2012, Nigeria. Abuja, 2012.

4. Moyo S ∙ Ismail F ∙ Van der Walt M ∙ et al. Prevalence of bacteriologically confirmed pulmonary tuberculosis in South Africa, 2017-19: a multistage, cluster-based, cross-sectional survey. *Lancet Infect Dis* 2022; **22**(8): 1172-80.

5. Elmadhoun WM ∙ Noor SK ∙ Bushara SO ∙ et al. Epidemiology of tuberculosis and evaluation of treatment outcomes in the national tuberculosis control programme, River Nile state, Sudan, 2011-2013. *East Mediterr Health J* 2016; **22**(2): 95-102.

6. Centers for Disease Control and Prevention (CDC) MUSoPHU, National Disease Control Division, Ministry of Health (Uganda).,. The Uganda National Tuberculosis Prevalence Survey, 2014-2015 Survey Report. Kampala, 2017.

7. Kapata N ∙ Chanda-Kapata P ∙ Ngosa W ∙ et al. The Prevalence of Tuberculosis in Zambia: Results from the First National TB Prevalence Survey, 2013-2014. *PLoS One* 2016; **11**(1): e0146392.

8. World Bank Group. Population, Total - Ghana, Lesotho, Nigeria, South Africa, Sudan, Uganda, Zambia. <https://data.worldbank.org/indicator/SP.POP.TOTL?locations=GH-LS-NG-ZA-SD-UG-ZM> (accessed Jan 10 2025).

9. Bates D ∙ Mächler M ∙ Bolker B ∙ Walker S. Fitting Linear Mixed-Effects Models Using lme4. *Journal of Statistical Software* 2015; **67**(1): 1 - 48.
